# Racial and Ethnic Disparities in Adolescent Combustible Tobacco Smoking From 2014 to 2020: Declines Are Lagging Among Non-Hispanic Black Youth

**DOI:** 10.1093/ntr/ntae001

**Published:** 2024-01-05

**Authors:** Dale S Mantey, Onyinye Omega-Njemnobi, LaTrice Montgomery, Steven H Kelder

**Affiliations:** Department of Health Promotion and Behavioral Science, UTHealth University of Texas School of Public Health, Houston, TX, USA; Michael and Susan Dell Center for Healthy Living, UTHealth School of Public Health, Austin Campus, Austin, TX, USA; Michael and Susan Dell Center for Healthy Living, UTHealth School of Public Health, Austin Campus, Austin, TX, USA; Department of Epidemiology, Human Genetics and Environmental Sciences, UTHealth University of Texas School of Public Health, Houston, TX, USA; Addiction Sciences Division, Department of Psychiatry and Behavioral Neuroscience, University of Cincinnati College of Medicine, Cincinnati, OH, USA; Michael and Susan Dell Center for Healthy Living, UTHealth School of Public Health, Austin Campus, Austin, TX, USA; Department of Epidemiology, Human Genetics and Environmental Sciences, UTHealth University of Texas School of Public Health, Houston, TX, USA

## Abstract

**Introduction:**

We quantified the linear trend in combustible tobacco smoking among adolescents in the United States from 2014 to 2020, and then compared these trends across racial and ethnic categories. We also tested the effect of e-cigarette use on these trends for all-youth and across racial and ethnic categories.

**Aims and Methods:**

We pooled and analyzed seven years of National Youth Tobacco Survey data for *n* = 124 151 middle and high school students from 2014 to 2020. Weighted logistic regression analyses calculated the annual change in combustible tobacco smoking (ie cigarettes, cigars, and hookah) from 2014 to 2020. Stratified analyses examined linear trends for non-Hispanic White (NHW), NH-Black (NHB), Hispanic/Latino, and NH-Other (NHO) youth. All-models controlled for sex, grade level, and past 30-day e-cigarette use.

**Results:**

Combustible tobacco smoking from 2014 to 2020 dropped by more than 50% for NHW youth, more than 40% for Latino and NHO youth, compared to just 16% among NHB youth. From 2014 to 2020, the odds of combustible tobacco smoking declined by 21.5% per year for NHWs, which was significantly greater than Hispanic/Latinos (17% per year; *p* = .025), NHOs (15.4% per year; *p* = .01), and NHBs (5.1% per year; *p* < .001), adjusting for sex, grade, and e-cigarette use. Trends and disparities in trends by race and ethnicity were observed independent of e-cigarette use.

**Conclusions:**

Combustible tobacco smoking declined for all-youth but at significantly different rates across races and ethnicities. Notably, declines in combustible tobacco smoking are lagging among NHB youth. Interventions are critically needed to address this disparity.

**Implications:**

A direct, evidence-based intervention to reduce combustible tobacco smoking among NHB youth is critically needed. Such tobacco control initiatives should follow the Best Practices for Comprehensive Tobacco Control Framework, incorporating sustainable funding for school-based intervention, public health education, and adult cessation.

## Introduction

Tobacco use among adolescents has evolved considerably over the past decade. Electronic cigarettes (e-cigarettes) have been the most used tobacco product among non-Hispanic White (NHW) and Hispanic/Latino youth from 2014 (~9.3%)^1^ to 2021 (~7.6%).^2^ Conversely, combustible cigars were the most used tobacco product among non-Hispanic Black (NHB) youth over this time,^1,2^ with the exception of 2019.^3^ Over this time, cigarette smoking declined significantly among youth across all-racial and ethnic categories.^1,2^ Combustible tobacco exposes the user to toxins and carcinogens as byproducts of combustion (eg carbon monoxide)^4,5^ that are not present in e-cigarettes.^4–8^ Additionally, combustible tobacco smoking has greater odds of nicotine dependence than e-cigarette use during adolescence.^9–12^ In other words, the most popular tobacco product among NHB youth (cigars) is significantly more dangerous and addictive than the most popular tobacco product among NHW and Hispanic youth (e-cigarettes) in the United States.

Shifting patterns of tobacco use requires an understanding of the tobacco retail environment^13^ and the commercial determinants of health.^14,15^ In the United States, the tobacco industry has a documented record of marketing its products to youth, particularly NHB youth.^16–19^ As noted by Garrett et al. (2016),^16^ the tobacco industry (eg Altria) has attempted to link their products with Black culture through direct advertisements of select brands of combustible cigarettes (eg Newport and Kool) and cigars (eg Black and Mild)^20,21^ in print and online. Similarly, price promotions (eg discounts at the point of sale) have been found to target zip codes with higher concentrations of NHB residents.^22^ The historical and contemporary context of the tobacco industry targeting NHB youth necessitates extensive and quantitative investigation.

e-Cigarette use is an important consideration in understanding trends in combustible tobacco smoking among youth in the United States.^6,8^ While decades of epidemiological study are required to fully understand the direct health consequences of e-cigarette use during adolescence,^6,8^ it is well-established that nicotine is an addictive, psychoactive stimulant, and neurotoxin for developing brains.^23–25^ Further, e-cigarettes have been linked to current (ie dual/poly use)^12,26–29^ and future combustible tobacco smoking among youth,^30–39^ though the causal pathway from e-cigarettes to combustible tobacco remains unclear.^34,40^

Evidence-based intervention and regulatory policy will be needed to address adolescent tobacco use, particularly combustible tobacco smoking. Further, research is required to explore racial and ethnic differences in combustible tobacco smoking and the cause of these disparities, considering the rise in e-cigarette use, to inform evidence-based public health intervention and regulatory policy. Subsequently, research on combustible tobacco smoking will need to account for e-cigarette use given the prevalence of e-cigarette use^1,2^ and the substantial overlap of e-cigarette use and combustible tobacco smoking (ie dual/poly use)^27–29^ among youth.

### Study Aims and Hypotheses

This study has three aims. First, we aim to examine combustible tobacco smoking trends among a nationally representative sample of middle and high school students from 2014 to 2020. Second, we aim to compare trends in combustible tobacco smoking across racial and ethnic categories: Non-Hispanic White (NHW); Non-Hispanic Black (NHB); Hispanic/Latino; and Non-Hispanic “Other” (NH-“Other”) from 2014 to 2020. Based on prior surveillance data, we hypothesize that combustible tobacco smoking will decline slower among non-Hispanic Black youth, relative to non-Hispanic White youth, from 2014 to 2020. Findings will provide insights into changing tobacco product use among youth and evaluate the degree of racial and ethnic disparities in these trends. Finally, we aim to examine the role of e-cigarette use as a possible effect modifier on combustible tobacco smoking trends from 2014 to 2020 for the entire sample and stratified by the four racial and ethnic categories.

## Methods

### Study Sample and Population

We pooled and analyzed seven years of National Youth Tobacco Survey (NYTS) data. The NYTS is an annual, cross-sectional survey of tobacco use behaviors among middle (sixth through eighth grade) and high (9th–12th grade) students in the United States. We did not use data from the 2021 NYTS surveys^41^ given methodologic changes related to the COVID-19 pandemic and our focus on examining reliable trends. The CDC explicitly states, “the 2021 NYTS estimates should not be compared with previous NYTS survey waves.”^41^

A total of *n* = 132 003 middle and high school students completed the NTYS from 2014 to 2020. Approximately 5.6% of the sample (*n* = 7852) had missing data on one or more study variables and were not included in the analytic model. The final sample was *n* = 124 151 middle and high school students from 2014 to 2020. We conducted a complete case analysis given our dataset’s missingness rate and size.^42^

### Measures

#### Past 30-Day Combustible Tobacco Smoking

The study outcome was past 30-day smoking of one or more combustible tobacco products: cigarettes, cigar products (ie large cigars, little filtered cigars, and cigarillos), or hookah. This study did not incorporate bidis, pipe tobacco, or roll-your-own. Any value greater than 0 indicated any past 30-day use. The NYTS methodology guide directed weighted estimates,^43^ applied to generate nationally representative prevalence data per year. We did not incorporate lesser-used tobacco products (eg bidis, pipe tobacco, or roll-your-own) in order to isolate analyses to more prevalent tobacco products among youth.

#### Annual Trend

The survey years were modeled as a continuous and independent variable. Years ranged from 2014 to 2020 and increased in units of one (ie 1 year). There were no gaps in the survey year.

#### Race and Ethnicity

This study examined trends in combustible tobacco smoking across racial and ethnic categories. Per CDC recommendations,^41^ participants were classified as: (1) non-Hispanic, White [NHW]; (2) non-Hispanic Black (NHB); (3) Hispanic/Latino; or (4) non-Hispanic “Other” [NHO], reflecting youth who identified as American Indian or Alaska Native, Asian, Native Hawaiian or Other Pacific Islanders, or any combination of race and ethnic categories.

#### Covariates

We controlled for sex; males served as the referents. We also controlled for grade level, with middle school (referent) indicating sixth–eighth grade students and high school indicating 9th 12th grade. Past 30-day e-cigarette use was incorporated as a covariate and possible effect modifier. Participants who reported using e-cigarettes on one or more days (of the past 30) were considered e-cigarette users.

### Statistical Analyses

First, we estimated the weighted prevalence of past 30-day combustible tobacco smoking by year and race/ethnicity for the total sample and stratified by past 30-day e-cigarette use (no/yes). Study hypotheses were tested using a series of multivariate logistic regression models. First, we conducted weighted, logistic regression models examining combustible tobacco smoking trends from 2014 to 2020 among middle and high school students, controlling for sex, grade level, race/ethnicity, and past 30-day e-cigarette use. Next, we modeled the interaction between year and race/ethnicity (ie syntax in Stata: c.year##i.race) to assess for differences in combustible tobacco smoking trends for NHB, Hispanic/Latino, and NHO youth relative to NHW. Then, weighted logistic regression analyses stratified by race/ethnicity were conducted to quantify the trends in combustible tobacco smoking from 2014 to 2020. Finally, we replicated the above analyses among youth who did (*n* = 14 383) and did not (*n* = 109 768) report past 30-day e-cigarette use; these analyses were conducted to account for the high rate of dual/poly tobacco product use among youth.^27–29^ Analyses were performed using Stata 14.2 (College Station, Texas). Sampling weights were applied in all-models to generate nationally representative estimates.

## Results

### Descriptive Statistics

As seen in Table 1, combustible tobacco smoking declined from 12.1% in 2014 to 6.6% in 2020 for the entire sample; trends are also reported by race/ethnic category. Demographic characteristics of the study sample are reported in Table 2.

**Table 1.** Descriptive Statistics Among Middle and High Students in the United States From 2014 to 2020

	Full sample	Non-Hispanic, White^a^	Non-Hispanic, Black^a^	Hispanic/Latino^a^	Non-Hispanic, Other^a^
	*N* = 124 151	*N* = 55 755	*N* = 17 454	*N* = 36 044	*N* = 14 898
	100%	52.3% (50.6–54.0)	12.7% (11.7–13.8)	24.3% (23.0–25.7)	10.7% (10.0–11.4)
Combustible tobacco smoking^b^					
No	90.7% (90.3–91.2)	90.8% (90.2–91.3)	90.6% (89.7–91.4)	90.3% (89.6–90.9)	91.8% (91.0–92.5)
Yes	9.3% (8.9–9.7)	9.3% (8.7–9.8)	9.5% (8.7–10.3)	9.7% (9.7–10.4)	8.2% (7.5–9.0)
Sex					
Male	50.5% (49.9–51.1)	50.7% (50.0–51.4)	50.4% (49.3–51.6)	50.1% (49.1–51.0)	50.7% (49.5–51.8)
Female	49.5% (48.9–50.1)	49.3% (48.6–50.0)	49.6% (48.4–50.7)	49.9% (49.0–50.9)	49.4% (48.2–50.5)
Grade level					
Middle school	43.3% (41.3–45.4)	42.2% (39.7–44.7)	43.9% (40.2–47.6)	45.3% (42.6–48.1)	43.8% (40.2–47.3)
High school	56.7% (54.6–58.7)	57.9% (55.3–60.3)	56.1% (52.4–59.8)	54.7% (52.0–57.4)	56.3% (52.7–59.8)
e-Cigarette use^c^					
No	87.9% (87.2–88.6)	86.1% (85.2–86.9)	93.6% (92.9–94.2)	88.1% (87.2–89.0)	89.7% (88.5–90.8)
Yes	12.1% (11.4–12.8)	13.9% (13.1–14.8)	6.4% (5.8–7.1)	11.9% (11.0–12.8)	10.3% (9.2–11.5)

^a^Per CDC recommendations, participants were classified as: (1) non-Hispanic White [NHW]; (2) non-Hispanic Black (NHB); (3) Hispanic/Latino; or (4) non-Hispanic “Other” [NHO], reflecting youth who identified as American Indian or Alaska Native, Asian, Native Hawaiian or Other Pacific Islanders or any combination of racial identities.

^b^Self-reported use of combustible cigarettes, cigar products, or hookah in the past 30 days.

^c^Self-reported use of electronic cigarettes in the past 30 days.

**Table 2.** Combustible Tobacco Smoking among Middle and High Students in the United States from 2014 to 2020.

	Full sample	Non-Hispanic, White^a^	Non-Hispanic, Black^a^	Hispanic/Latino^a^	Non-Hispanic, Other^a^
Combustible Tobacco Smoking Prevalence^b^					
2014	12.1% (11.2–13.1)	11.9% (10.6–13.3)	11.1% (8.2–11.5)	14.7% (13.1–16.6)	11.2% (9.4–13.3)
2015	11.3% (10.0–12.7)	11.0% (9.4–12.9)	11.1% (8.3–14.6)	12.3% (10.8–14.0)	11.9% (9.9–14.3)
2016	9.4% (8.5–10.4)	9.6% (8.3–11.0)	9.6% (8.3–11.1)	9.7% (8.5–11.0)	8.3% (6.7–10.3)
2017	8.6% (7.5 -9.7)	9.2% (7.9–10.6)	7.7% (6.2–9.5)	8.6% (7.1–10.5)	6.9% (5.2–9.1)
2018	9.0% (8.1 -10.1)	9.3% (8.1–10.8)	8.7% (7.0–10.7)	9.3% (8.2–9.6)	7.7% (6.2–19.5)
2019	8.6% (7.6–9.8)	8.2% (6.9–9.9)	11.6% (9.4–14.3)	8.5% (7.4–9.7)	7.6% (5.9–9.7)
2020	6.6% (5.6–7.7)	5.6% (4.4–7.1)	8.6% (7.1–10.3)	7.9% (6.3–10.0)	6.3% (4.6–8.56)

^a^Per CDC recommendations, participants were classified as: (1) non-Hispanic, White [NHW]; (2) non-Hispanic Black (NHB); (3) Hispanic/Latino; or (4) non-Hispanic “Other” [NHO], reflecting youth who identified as American Indian or Alaska Native, Asian, Native Hawaiian or Other Pacific Islanders or any combination of racial identities.

^b^Self-reported use of combustible cigarettes, cigar products, or hookah in the past 30-days.

### Trends in Combustible Tobacco Smoking Among Youth

We found that the odds of combustible tobacco smoking significantly declined each year from 2014 to 2020 among all-middle and high school students in the United States (aOR: 0.83; 95% CI = .81 to .85), adjusting for sex, race/ethnicity, grade level, and past 30-day e-cigarette use. However, these trends in combustible tobacco smoking differed significantly by race/ethnicity (Table S1).

Stratified analyses found that the odds of combustible tobacco smoking significantly declined each year among NHW (aOR: 0.78; 95% CI = .76 to .81), Hispanic/Latino (aOR: 0.83; 0.80 – 0.86), NHO (aOR: 0.85; 95% CI = .80 to .89), and NHB (aOR: 0.95; 95% CI = .91 to .99). Each analysis controlled for sex, grade level, and past 30-day e-cigarette use. Analyses for the full sample and stratified by race/ethnic category are provided in Table 3.

**Table 3.** Trend in Combustible Tobacco Smoking Prevalence by Race/Ethnicity for Middle and High School Students From 2014 to 2020

	Middle and high school students	Non-Hispanic, White^a^	Non-Hispanic, Black^a^	Hispanic/Latino^a^	Non-Hispanic, Other^a^
	Adjusted OR (95% CI)	Adjusted OR (95% CI)	Adjusted OR (95% CI)	Adjusted OR (95% CI)	Adjusted OR (95% C I)
Linear trends					
Year (2014–2020)	0.83*** (0.81–0.85)	0.78*** (0.76–0.81)	0.95* (0.91–0.99)	0.83*** (0.80–0.86)	0.85*** (0.80–0.89)
Sex					
Male	1.00 (Referent)	1.00 (Referent)	1.00 (Referent)	1.00 (Referent)	1.00 (Referent)
Female	0.91** (0.85–0.96)	0.79*** (0.76–0.81)	1.09 (0.94–1.25)	1.05 (0.94–1.17)	0.95 (0.78–1.16)
Grade level					
Middle school	1.00 (Referent)	1.00 (Referent)	1.00 (Referent)	1.00 (Referent)	1.00 (Referent)
High school	2.74*** (2.50–3.00)	3.44*** (2.98–3.97)	3.13*** (2.59–3.80)	1.97*** (1.73–2.23)	2.37*** (1.91–2.96)
e-Cigarette use^c^					
No	1.00 (Referent)	1.00 (Referent)	1.00 (Referent)	1.00 (Referent)	1.00 (Referent)
Yes	15.36*** (14.28–16.53)	15.86*** (14.32–17.55)	12.17*** (10.08–14.69)	16.69*** (14.75–18.88)	14.33*** (11.58–17.73)
Race/ethnicity^a^		N/A	N/A	N/A	N/A
Non-Hispanic White	1.00 (Referent)	–	–	–	–
Non-Hispanic Black	1.65*** (1.47–1.84)	–	–	–	–
Non-Hispanic “Other”	1.11 (0.99–1.24)	–	–	–	–
Hispanic	1.31*** (1.20–1.42)	–	–	–	–

***p < .001; **p < .01; *p <.05.

### Non-e-Cigarette Users

As seen in Table 4, we found that the odds of combustible tobacco smoking significantly declined each year among non-e-cigarette users (aOR: 0.85; 95% CI = .82 to .87), adjusting for sex, race/ethnicity, and grade level. Analyses of non-e-cigarette users stratified by race/ethnicity found that the odds of combustible tobacco smoking significantly declined each year among NHW youth (aOR: 0.81; 95% CI = .78 to .84), Hispanic/Latino (aOR: 0.83; 0.79 – 0.87), and NHO (aOR: 0.86; 95% CI = .81 to .92) youth who did not use e-cigarettes. However, no decline in combustible tobacco smoking was observed from 2014 to 2020 among NHB youth who did not use e-cigarettes (aOR: 0.96; 95% CI = .91 to 1.00). Each analysis controlled for sex and grade level.

**Table 4.** Trend in Combustible Tobacco Smoking Prevalence by Race/Ethnicity for Middle and High School Students From 2014 to 2020, Stratified by Current e-Cigarette Use Status

	Middle and high school students	Non-Hispanic, White^a^	Non-Hispanic, Black^a^	Hispanic/Latino^a^	Non-Hispanic, Other^a^
	Adjusted OR (95% CI)	Adjusted OR (95% CI)	Adjusted OR (95% CI)	Adjusted OR (95% CI)	Adjusted OR (95% CI)
Non-e-cigarette users					
Year (2014–2020)	0.85*** (0.82–0.87)	0.81*** (0.78–0.84)	0.96 (0.91–1.00)	0.83*** (0.79–0.87)	0.86*** (0.81–0.92)
					
e-Cigarette users					
Year (2014–2020)	0.81*** (0.78–0.83)	0.77*** (0.74–0.80)	0.97 (0.89–1.05)	0.84*** (0.80–0.88)	0.83*** (0.77–0.90)

***p < .001; **p < .01; *p <.05.

Models adjust for sex and grade level. The analyses for the full sample also controlled for race/ethnicity.

### e-Cigarette Users

As seen in Table 4, we found that the odds of combustible tobacco smoking significantly declined each year among current e-cigarette users (aOR: 0.81; 95% CI = .78 to .83), adjusting for sex, race/ethnicity, and grade level. Analyses stratified by race/ethnicity found that the odds of combustible tobacco smoking significantly declined each year among NHW youth (aOR: 0.77; 95% CI = .74 to .80), Hispanic/Latino (aOR: 0.84; 0.80–0.88), and NHO (aOR: 0.83; 95% CI = .77 to .90) youth who used e-cigarettes. However, no decline in combustible tobacco smoking was observed from 2014 to 2020 among NHB youth who used e-cigarettes (aOR: 0.97; 95% CI = .89 to 1.05). Each analysis controlled for sex and grade level.

## Discussion

This study compared trends in combustible tobacco smoking by race/ethnic category among middle and high school students in the United States. We found odds of combustible tobacco smoking declined 21.5% per year among NHW youth from 2014 to 2020, which was 4.22 times greater than NHBs (5.1% per year), ~1.39 times greater than NHOs (15.4%), and ~1.26 times greater than Hispanic/Latinos (17.0%). Racial/ethnic disparities in combustible tobacco smoking trends from 2014 to 2020 were observed independent of e-cigarette use. These figures show that the reductions in adolescent tobacco use observed since 2014 have not equitably reached communities of color, particularly non-Hispanic-Black youth.

This study has several implications for public health research and practice. From a regulatory perspective, our findings indicate a need to address the commercial and social determinants of health that have resulted in disparate trends in combustible tobacco smoking among NHB youth in the United States. For example, adolescent tobacco use is heavily influenced by exposure to direct advertising of tobacco.^5–7^ There is an established racial and ethnic bias in tobacco marketing resulting in racial and ethnic differences in cigarette brand preferences.^18,44–47^ Historically, the tobacco industry has focused on cigarettes as the primary mode of nicotine delivery, with marketing efforts designed to promote differing brands (eg Newport and Camel) and product characteristics (eg menthol) and tailored this marketing by race and ethnicity.^18,44–47^ At present, less is known about the tactics and expenditures of tobacco industry marketing for non-cigarette, combustible tobacco products (cigars; hookah) and e-cigarettes.^48,49^ Our findings raise concerns about racial and ethnic biases in the marketing of combustible tobacco products (cigars; hookah) that may require regulatory oversight. These concerns are extended to the marketing of e-cigarettes, which is linked to e-cigarette use^50,51^ and remains largely unregulated or systematically monitored.^48,49^ The shifting behaviors in adolescent tobacco use suggest the need to research and, if necessary, address the marketing of specific tobacco products like cigars and e-cigarettes in line with combustible cigarettes to address adolescent tobacco use, including dual/poly use.

This study also has findings for tobacco prevention efforts and interventions. To date, tobacco prevention programs have largely focused on combustible cigarettes^7^ or e-cigarettes.^52,53^ School-based interventions have been demonstrated to effectively reduce adolescent tobacco initiation by up to 12% per year.^54,55^ As such, school-based interventions tailored to NHB youth and cigar smoking have the potential to address the 4:1 ratio in combustible tobacco declines observed in this study, provided these efforts receive appropriate investment and resources. Such interventions should be informed by population-based research through participatory community methods that incorporate the intervention stakeholders (eg youth, parents, school staff, and community) into the development, pre/pilot testing, implementation, dissemination, and evaluation of school-based interventions tailored to racially minor youth.

It is important to note that the two most commonly used tobacco products in this study from 2014 to 2021 among all-youth (ie e-cigarettes; cigars) are also two of the most popular modalities for cannabis use among youth. Specifically, e-cigarettes are the most used tobacco product and the fastest-growing modality of cannabis use among youth in the United States.^56^ Similarly, blunts (ie, hollowed-out cigars filled with cannabis flowers) are uniquely popular as a modality for cannabis use among NHB youth.^57^ The NYTS does not assess for cannabis use via blunts or e-cigarettes and, as a result, we cannot account for cannabis use in the observed trends. Future studies will need to explore the impact of cannabis use via blunt smoking and cannabis vaping on the trends reported in this study.

Published data suggest that cigar products account for a sizable proportion of the disparities in declines of combustible tobacco smoking. However, future research will need to consider the complexities of multiple tobacco product use when further examining the trends observed in this study. In the United States, multiple tobacco product use is most commonly characterized by the use of e-cigarettes and one (or more) combustible tobacco products.^12,26^ Our examination of trends in combustible tobacco smoking accounted for differences by e-cigarette use (ie the most used tobacco product among youth); however, additional study will be needed to investigate trends and disparities in youth smoking of multiple combustible products (eg cigarettes and cigars).

This study has several limitations. First, data on economic factors was not available for this analysis. In the United States, economic indicators (eg income; health insurance) are consistent predictors of tobacco use and nicotine dependence^.57^ Youth from low-income backgrounds are also exposed to a greater proportion of risk factors for tobacco use, such as tobacco retail marketing, price promotions, and outlet density.^58^ Future research must consider economic factors when attempting to generalize or replicate the findings presented in our study. Second, our analyses categorized race/ethnicity based on federal standards set by the Office of Management and Budget (OMB) 1997 Statistical Policy Directive No. 15: Standards for Maintaining, Collecting, and Presenting Federal Data on Race and Ethnicity.^59^ However, race and ethnicity are not inherently discrete categories but are, instead, social constructs created and shaped through political and economic considerations.^60^ Our operationalization of race/ethnic category, based on federal guidelines, inhibits the findings of this study from informing on the nuances of racial and ethnic identities in the United States. Third, we could not account for tobacco and cannabis couse via e-cigarettes (ie vaping) or cigars (ie blunt smoking). As a result, it is plausible that a proportion of the combustible tobacco smokers were blunt smokers or e-cigarette users vaping cannabis (rather than nicotine). While this may bias the study by inflating the number of combustible tobacco users and/or e-cigarette users, cannabis users who abstain from nicotine remain relevant to understanding tobacco use patterns among youth. Specifically, adolescents who vape cannabis or smoke blunts are still obtaining and using a tobacco product modified for cannabis use (ie blunts or e-cigarettes). Fourth, our findings are nationally representative and may not necessarily generalize to small units (eg states; and cities). Fifth, this study did not account for smokeless tobacco use, given the low prevalence and potential for collinearity in a large, nationally representative dataset. Nuanced, regional studies should explore the relationships observed in this study for smokeless tobacco use among relevant populations.

To summarize, this study has broad implications for public health intervention, research, and regulatory policy, despite the limitations of cross-sectional, observational data. The substantial declines in combustible tobacco smoking among adolescents from 2014 to 2020 were not observed among NHB youth. While combustible tobacco smoking did decline modestly (16%) for NHB youth who did not use e-cigarettes, combustible tobacco smoking was essentially cut in half for all-other youth independent of e-cigarette use, from 2014 to 2020. A direct, evidence-based intervention aimed at reducing combustible tobacco smoking among NHB youth is essential and should follow evidence-based methodologies that incorporate sustainable funding for school-based intervention, public health education, and adult cessation treatment.^4–7^

## Supplementary material

Supplementary material is available at *Nicotine and Tobacco Research* online.

ntae001_suppl_Supplementary_Table_1

## Data Availability

The data underlying this article are publicly available from the Centers for Disease Control and Prevention (CDC). The datasets were derived from sources in the public domain: https://www.cdc.gov/tobacco/data_statistics/surveys/nyts/index.htm
