# Supplementary material for: Racial and Ethnic Disparities in Adolescent Combustible Tobacco Smoking From 2014 to 2020: Declines Are Lagging Among Non-Hispanic Black Youth
Source: Nicotine Tob Res. 2024 Jan 5;26(7):940–7. doi: 10.1093/ntr/ntae001 (PMC11190047; doi:10.1093/ntr/ntae001)
Supplement: ntae001_suppl_Supplementary_Table_1 [file ntae001_suppl_supplementary_table_1.docx]

|  | Current Combustible Tobacco Use |
| --- | --- |
|  | Adjusted Odds Ratio  (95% Confidence Interval) |
| **Linear Trends * Race/Ethnicity**^a^ |  |
| Year * Non-Hispanic White | 1.00  (Referent) |
| Year * Non-Hispanic Black | 1.19***  (1.13 – 1.26) |
| Year * Non-Hispanic “Other” | 1.07*  (1.01 – 1.13) |
| Year * Hispanic | 1.05*  (1.01 – 1.10) |
|  |  |

Supplemental Table 1: Interaction effect between Survey Year and Race/Ethnicity.

The interaction effect was continuous by categorical (c.year##i.race)
